# PACS for Bhutan: a cost effective open source architecture for emerging countries

**DOI:** 10.1007/s13244-016-0512-7

**Published:** 2016-07-28

**Authors:** Osman Ratib, Nicolas Roduit, Dechen Nidup, Gerard De Geer, Antoine Rosset, Antoine Geissbuhler

**Affiliations:** 1 University Hospital of Geneva, Geneve, Switzerland; 2 Jigme Dorji Wangchuck National Referral Hospital, Thimphu, Bhutan; 3 Imagerie & Développement (ID), Geneva, Switzerland; 4 La Tour Réseau de Soins, Geneva, Switzerland; 5 Department of Medical Imaging and Information Sciences, Division of Nuclear Medicine, Rue Gabrielle-Perret-Gentil 4, 1211 Geneve 14, Switzerland

**Keywords:** Medical imaging, Picture archiving and communication systems, Radiology information system, Teleradiology, Software tools

## Abstract

**Abstract:**

This paper reports the design and implementation of an innovative and cost-effective imaging management infrastructure suitable for radiology centres in emerging countries. It was implemented in the main referring hospital of Bhutan equipped with a CT, an MRI, digital radiology, and a suite of several ultrasound units. They lacked the necessary informatics infrastructure for image archiving and interpretation and needed a system for distribution of images to clinical wards.

The solution developed for this project combines several open source software platforms in a robust and versatile archiving and communication system connected to analysis workstations equipped with a FDA-certified version of the highly popular Open-Source software. The whole system was implemented on standard off-the-shelf hardware.

The system was installed in three days, and training of the radiologists as well as the technical and IT staff was provided onsite to ensure full ownership of the system by the local team. Radiologists were rapidly capable of reading and interpreting studies on the diagnostic workstations, which had a significant benefit on their workflow and ability to perform diagnostic tasks more efficiently. Furthermore, images were also made available to several clinical units on standard desktop computers through a web-based viewer.

***Messages/teaching points*:**

• *Open source imaging informatics platforms can provide cost-effective alternatives for PACS*

• *Robust and cost-effective open architecture can provide adequate solutions for emerging countries*

• *Imaging informatics is often lacking in hospitals equipped with digital modalities*

## Introduction

Digital imaging modalities are becoming more widely available even in remote areas of emerging countries extending the capabilities of these areas to provide higher quality of care and diagnostic capabilities locally. While most of these centres can afford state-of-the-art imaging equipment, they often lack the needed IT infrastructures to manage and distribute the digital imaging data. Adding these informatics infrastructures will add a significant cost in the context of limited budgets, assigning priorities to medical equipment needed for patient care and for diagnostic services. This often leaves the users and care providers with limited resources for proper management of digital imaging data. Furthermore, efficient image interpretation requires specific software tools and imaging platforms that radiologists need for their daily task of image interpretation and reporting.

In Bhutan, a small kingdom of a little over 700000 inhabitants south of the chain of the Himalayans, the Jigme Dorji Wangchuck National Referral Hospital in Thimphu, the capital, is the only radiology centre equipped with a CT scanner, MRI scanner, digital radiology unit, and a suite of five ultrasound units. In a first informal visit to the hospital and discussion with local radiologists, we identified that they lack the proper infrastructure to manage and review the imaging data acquired from these imaging modalities. Images are interpreted directly on the scanner consoles located in the imaging suites, competing with the technologist’s task of performing imaging procedures concurrently. Besides the physical dispersion of these devices requiring radiologists to move from one unit to another to review and interpret the studies, it has a significant impact on the workflow and efficiency of radiologists in their daily tasks. Furthermore, the lack of proper storage and archiving of the images requires saving digital data on individual CDs that are often hard to retrieve when a patient returns for a new study and the need for comparing with previous studies is required. Finally, there is an increasing need for facilitating the communication between radiologists and referring physicians and clinical units in the hospital. Distribution of medical images to clinical wards and directly to clinical partners is particularly difficult given that only a very limited number of images are being printed for economic reasons, reading images from CDs remains limited and cumbersome for referring physicians. While the hospital is equipped with a secure local area network, it lacks the proper software and infrastructure to provide access to imaging data in a cost effective and easy way.

In recent years a trend toward development of open source software platforms in medicine have made great progress toward providing very robust and reliable alternative solutions to commercial products [1]. Several open source solutions for image display and analysis are available. We have contributed in over 10 years now to the development and evolution of the OsiriX imaging platform [2], a widely adopted image review and analysis software that is used by radiologists and clinicians around the world for interpretation of all kinds of digital images [3]. Several spin-offs of certified versions of the OsiriX software are now available from different vendors. Through a non-profit foundation we continue to encourage and sponsor young developers as well as innovative initiatives based on open source platforms [4, 5].

## Material and methods

The department of radiology at Wangchuck National Referral Hospital is equipped with a CT scanner (Brilliance 16 from Philips Medical), MRI scanner (Signa 1.5 T from General Electric), and a digital radiology unit (IDC DR 1590 CURA) as well as five ultrasound units from different vendors (two Logiq e series GE Medical, one Siemens Acuson X500, and two Philips Clear Vue 350). All these modalities have the ability to export images digitally in DICOM compliant format. The hospital is also equipped with an internal secured Ethernet local area network available across the hospital as well as a restricted Wi-Fi network. The radiology department benefits from a separate sub-network allowing a better communication bandwidth between different imaging equipment.

The PACS server was implemented on a Supermicro 7048R-TR server running under the Linux operating system. The server specifications included:

- 1× Intel Xeon Processor E5-2650 v3 (25 M Cache, 2.30 GHz)
- 2× DDR4 16GB 2133MHz (memory)
- 2× 240GB HyperX Predator PCIe (SSD card)
- 8× WD Green 2 TB Desktop SATA 3.5”
- 2× RJ45 Gigabit Ethernet LAN ports 1× RJ45
- 2× 920 W redundant power supplies

The software framework consisted of the following components:

- Operating system: openmediavault (http://www.openmediavault.org), a popular Linux distribution for an open network attached storage solution. This distribution is based on Linux Debian and provides a Web-based Graphical User Interface (Web-GUI).
- The open source dcm4che DICOM archive solution with a MySQL database (http://dcm4che.org). A specific Debian package has been built to facilitate the installation directly through the Web-GUI as a service of openmediavault (https://github.com/nroduit/openmediavault-dcm4chee). Part of this work is based on the open source CDMEDIC solution (http://cdmedicpacsweb.sourceforge.net) by Dr. Pablo Sau, Valencia.
- Java-based web viewer (Weasis developed by Nicolas Roduit – Geneva), http://dcm4che.atlassian.net/wiki/display/WEA/Home)
- Advanced image analysis platform (OsiriX developed by Dr. Antoine Rosset and the OsiriX team - Geneva)

The PACS service component allows managing and enabling/disabling the PACS server through a dedicated Web-based graphic user interface (GUI). Likewise, the PACS activity can be monitored directly from the Web-GUI.

The operating system is installed on the SSD disks, which are mirrored by a RAID-1 configuration, while all the archived images are stored in a Raid-5 virtual partition (six hotswap disks and two hotspare disks).

**Fig. 1:**
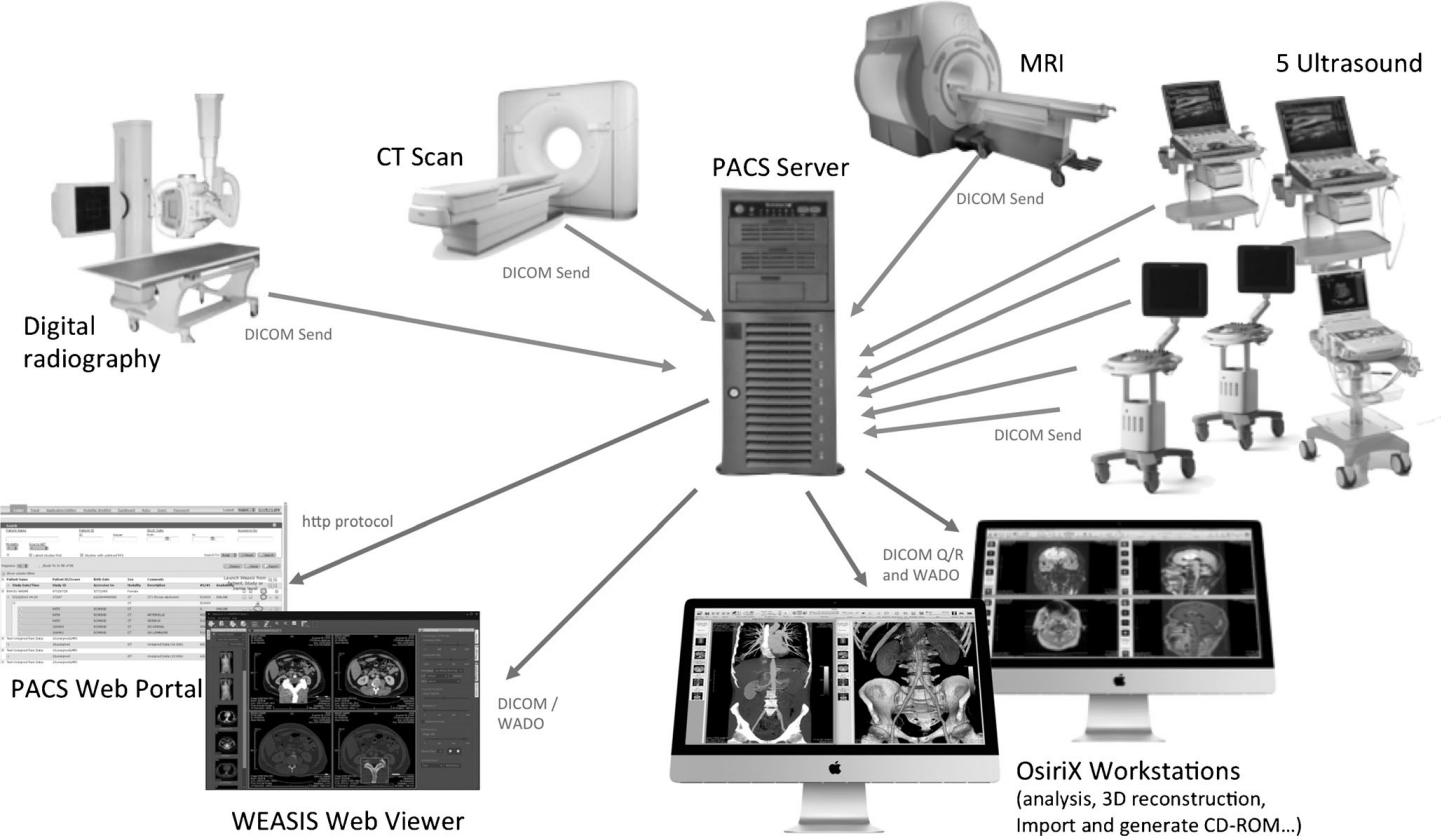
The general diagram of the imaging network associated with the PACS server that was implemented showing the different imaging modalities, the primary interpretation workstations, and web-based applications that can run on any standard desktop computer

Two workstations based on 27” retina display iMac workstations from Apple Computer are equipped with DICOM-compliant OsiriX software for diagnostic interpretation and image analysis. The general diagram of the system is shown in Fig. 1.

The volumes of images generated per year by the different imaging modalities are listed in Table 1. In addition to the three main digital imaging modalities, there is a volume of selected images and dynamic sequences from ultrasound examinations that are being archived for reference and follow-up of patients. The storage volume of the latter is hard to estimate given that only selected studies are archived on demand.

**Table 1:** Estimated volumes of image data generated by the three main digital imaging modalities based on the current number of studies performed

Modality	Studies/year	MB/study	Est Total vol. (GB)
CT	6000	250	1500
MR	4000	40	160
Dig RX	5000	15	75
Total			1735

These volumes are based on uncompressed data; lossless compression can reduce these volumes by half

The clinical staff of the radiology departments consist of three board certified radiologists, two radiology technologists (with bachelor's degrees), 14 radiographers (certificate degree), eight ultrasound technicians (diploma and certificate degree), four CT technicians (diploma and certificate degree), three MRI technicians (diploma and certificate degrees) as well as support from a local IT team for support and management of IT infrastructure.

While the hospital is equipped with a secure intranet local area network, it does not offer any electronic patient record or digital hospital information system. Likewise, the radiology department is not yet equipped with a radiology information system (RIS) and all orders for radiological procedures as well as radiology reports are handled in paper format. While OsiriX software supports the option of importing orders and generating diagnostic reports, it was implemented as a stand-alone PACS network for image viewing and analysis. It is, however, fully compatible with future deployment of RIS and HIS systems.

## Results and observations

**Fig. 2:**
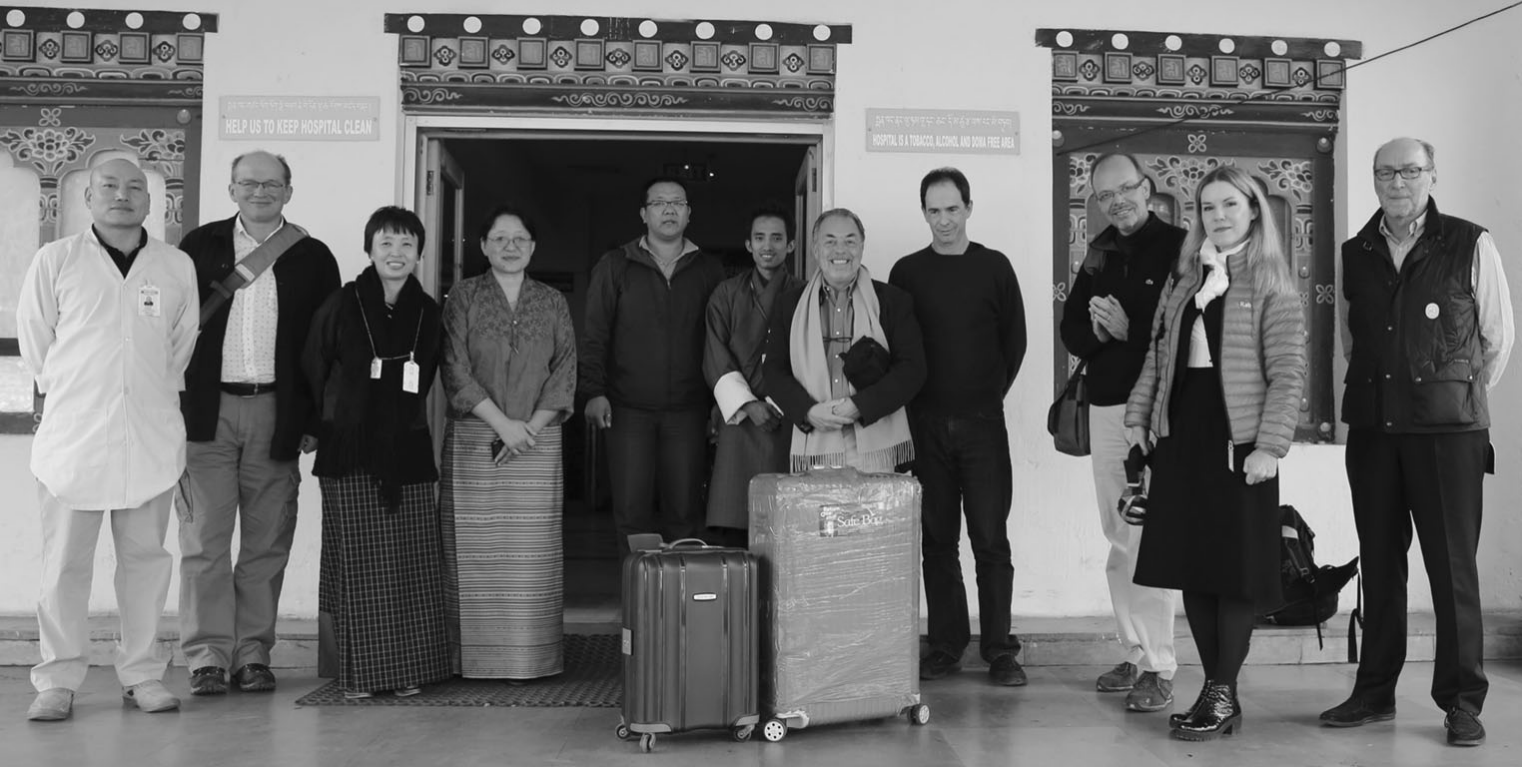
The Geneva team delivering the system was greeted by the local team at arrival in the Bhutan hospital

A complete pre-configured server unit with all software components installed and tested was transported to Bhutan and installed by the Geneva team in collaboration with the local team (see Fig. 2). Technical support engineers from the different imaging equipment companies were available on site or remotely through phone communication for configuring the imaging modalities to connect to the PACS server and viewing workstations through standard DICOM protocols. Proper preparation and setup of the network and equipment identities was carried out prior to our arrival by the local IT team.

**Fig. 3:**
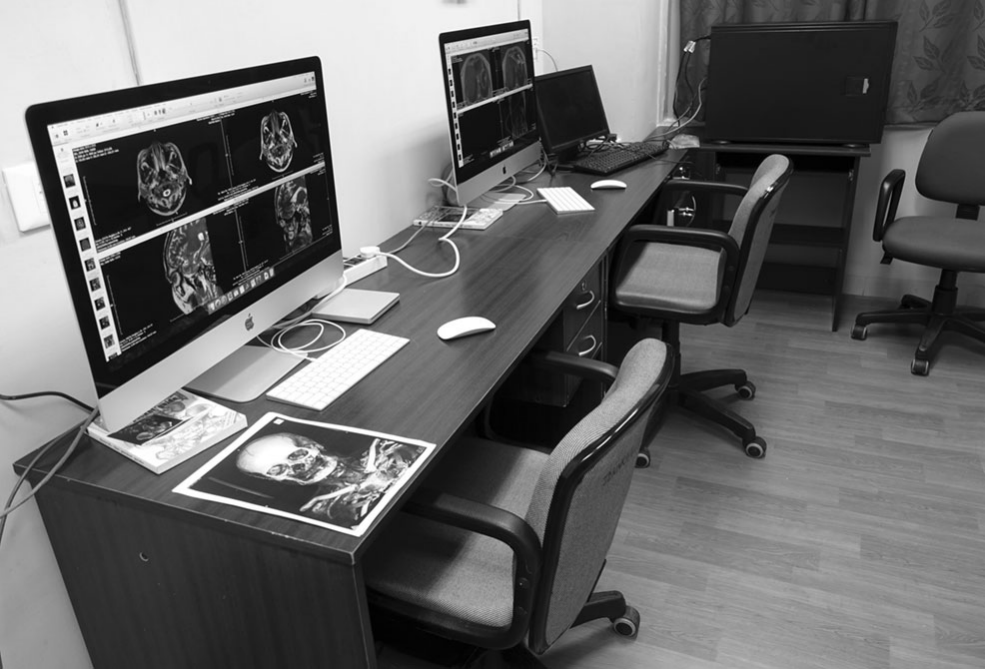
The setup of the radiology reading room after installation of the two OsiriX workstations

A special room was set up to serve as a reading room for radiologists where the two OsiriX workstations were installed (see Fig. 3). The full installation of the complete system was carried out in less than 48 h, followed by appropriate training of the technical and IT staff as well as the radiologists in managing and using the system. A PACS manager was designated among the hospital IT team and was trained for management, updates, and backup tasks of the system. The lead technologists of each modality were trained to monitor and perform regular quality control tasks of the images archived on the system. Remote support of the IT and technical teams was also provided by our team in Geneva. The ease of use and well-known user interface of the OsiriX software allowed radiologists to rapidly become familiar with its main features and adopt it for retrieving and reviewing studies from all the imaging modalities.

**Fig. 4:**
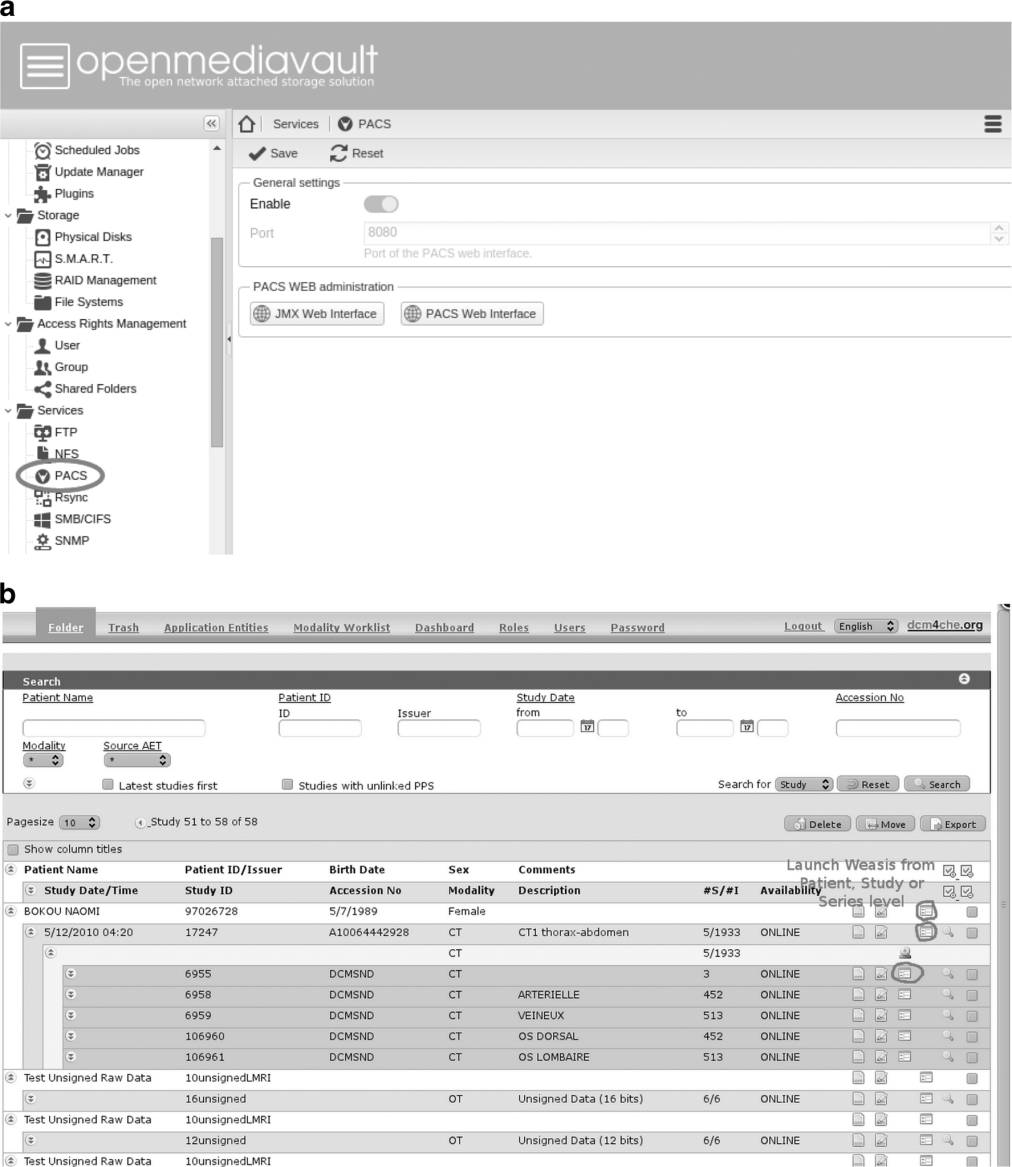
**a** The main window of the “OpenMediaVault” software used for data management. This basic framework was extended with a specific “PACS” plugin designed for management of DICOM files and medical imaging data, **b** The management and basic maintenance functions of the PACS server are accessible through a simple user-friendly web interface that is accessible remotely to the IT support team as well as to the radiology technologists

**Fig. 5:**
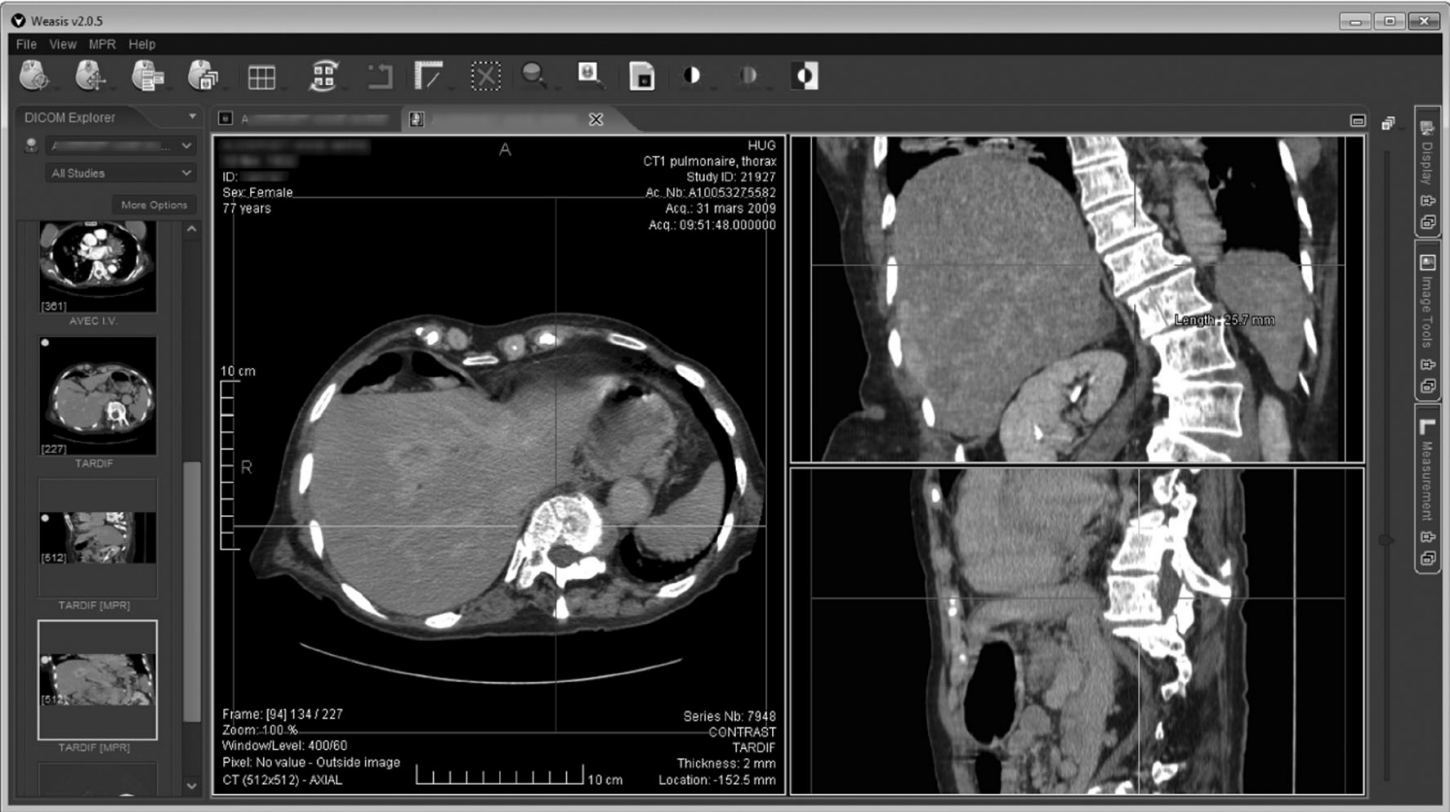
A screenshot of the Web-based image viewer (WEASIS) than can be deployed across the hospital allowing access to images from any workstation in the clinical wards

The management of the PACS server as well as monitoring and supervision of the performance of the system is available through a simple Web-GUI that was partially developed by our team specifically for this project. This allows technologists of the radiology department, as well as members of the IT team, to remotely access the server and perform the basic management and maintenance tasks. This user-friendly platform allows technologists to easily correct errors that may occur in demographic data and image identification data. The main window of the web-portal to the PACS server is shown in Fig. 4. It was implemented as an extension of an open source data storage platform (openmediavault) with an extended set of features and data management components that are accessible through a simple graphic user interface, Fig. 5.

Furthermore, the IT staff was trained to extend the access to images to clinical wards through a web-based viewer that can easily be launched on conventional desktop computers around the hospital through standard web browsers. The only requirement is the installation at the first launch of the Java Runtime Environment (JRE) on the local operating system. The first units that were connected to the PACS were the emergency unit and the intensive care unit. Other workstations in physicians' and surgeons' offices were also set up. This was progressively extended to different locations in the hospital without any additional cost or hardware.

At the time of submission of this paper, the system was operational without any interruption for over 3 months and the local IT team was in charge of management and maintenance of the system performing regular backups and reporting of the system performance. The technologists and the radiologists were fully autonomous in using the system in clinical routines. All studies of the digital modalities of CT, MRI, and digital radiography were archived automatically on the PACS server, while selected sets of images from the different ultrasound units were archived and routed to the diagnostic workstation on demand.

## Discussion and conclusions

This pilot project of implementing a robust and cost-effective solution for image management and interpretation allowed establishing a model of a system that can easily be disseminated in a large number of institutions around the world that already have digital imaging modalities but cannot afford the purchase and implementation of image management infrastructure and PACS systems. The PACS server architecture that we developed based on existing open source components can easily be replicated on standard off-the-shelf hardware [6]. The global package is made available as free open source software by us on the publicly accessible server (https://github.com/nroduit/openmediavault-dcm4chee). The system is also fully compliant with other commercial platforms of radiology information systems (RIS) or electronic patient records in hospital-wide information systems (HIS). The dedicated high performance OsiriX platform allowing advance image processing and visualization is complemented by a web-based viewer (WEASIS) that is compatible with all operating systems [7]. This architecture follows a general trend in imaging informatics aiming toward the development of open source components in an open architecture that can easily be expandable [8]. It can also be extended to wireless devices and tablets through WiFi networks using existing mobile DICOM viewers.

The main feature of the system is its simple architecture that facilitates the adoption by local teams that can easily be trained to use it, but also to manage it and extend it to adapt it to the evolving needs of the hospital. New modalities can easily be added to the network with simple procedures for configuration of the PACS server. The goal being that each institution should take ownership of such a system and be directly involved in its operation. We will continue to provide the necessary technical support and training for the local teams to become fully independent and autonomous in maintaining the system. In its current implementation, the PACS system does not benefit from a RIS or HIS companion system. To benefit from more efficient and complete imaging workflow, integration of a RIS could provide the additional features of management and distribution of radiological reports. The open source software components allow implementing the system on any off-the-shelf hardware. The system is also fully compliant with IHE (Integrated Healthcare Enterprise www.ihe.net) profiles allowing easy integration with future hospital information systems (HIS and RIS) and can easily link to electronic medical records through a basic set of web services. It also offers a flexible platform for teleradiology as well as for setting up anonymized teaching and education databases.
